# Late Gadolinium Enhancement does occur in the Tako-Tsubo Cardiomyopathy - a quantitative cardiovascular magnetic resonance study

**DOI:** 10.1186/1532-429X-13-S1-P314

**Published:** 2011-02-02

**Authors:** Christian Hamilton-Craig, Kris Nowakowski, Nick J Brett, Wendy E Strugnell, Richard E Slaughter

**Affiliations:** 1 Centre of Excellence in Cardiovascular MRI, Brisbane, Australia; 2 Department of Cardiology, Prince Charles Hospital, Brisbane, Australia

## Background

Tako-tsubo cardiomyopathy (TTC) accounts for 1% of admissions for chest pain in Japan and up to 1 in 30 cases referred for acute primary angioplasty in Western cohorts. The absence of myocardial late gadolinium enhancement (LGE) on cardiac magnetic resonance imaging (CMR) has been recommended in the literature to distinguish TTC from either acute myocardial infarction or myocarditis.

## Method

Data were retrospectively reviewed from 27 consecutive patients with confirmed Mayo Clinic criteria for TTC (clinical, ECG, angiographic and left ventriculographic findings) presenting to our institution over a 2 year period. A CMR (GE 1.5T Twinspeed) protocol of steady state free precession, triple inversion recovery, and myocardial LGE imaging was performed in the acute phase. LGE was quantified using standardized software (ReportCard V4.0), with both 2 and 5 standard deviations thresholds (SD) above normal remote myocardium, normalized to LV mass.

## Findings

**Figure 1:**
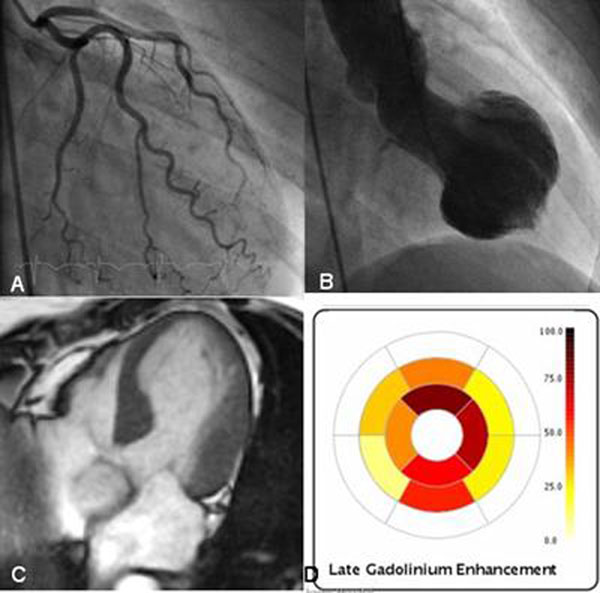
A) angiography, B) ventriculography, C) systolic SSFP 4chamber frame, D) quantitative LGE plot.

All patients had raised serum troponin I (mean 3.7 ug/mL, range 0.2-30). Mean ejection fraction by CMR was 43% ±8.5% (range 25-51%), mean door-to-CMR time 59 hours. All patients had significantly abnormal wall motion; apical ballooning in 18 and midwall variant in 9 cases. Eleven patients (41%) had diffuse LGE localized to the area of abnormal wall motion, representing 34.5 ±9% LV mass. In 9 of these 11 patients, LGE signal intensity was >5 SD above normal myocardium, representing 16.5 ±12% LV mass. This has not been previously described. Those with >5SD LGE had raised LV volumes and reduced EF (p=0.047). Mean troponin trended to higher levels in LGE positive patients (2.31±1.8 vs 4.69±8.6, p=0.10). The pattern of LGE differed to that seen in both ischemia and myocarditis. Improvement in segmental function was confirmed by follow-up CMR or echocardiography in all patients. Figure 1

## Conclusion

In this single-center series, LGE was present in 41% of cases of TTC, occurring in a diffuse pattern through the areas of myocardial stunning, 82% of which were >5SD above normal myocardium. This likely represents diffuse myocyte damage. Absence of LGE should not, therefore, be used as a diagnostic criterion for TTC. LGE does occur in the tako-tsubo cardiomyopathy, and presence of intense (>5SD) LGE appears associated with worse myocardial injury.

